# Addressing grading bias in rock climbing: machine and deep learning approaches

**DOI:** 10.3389/fspor.2024.1512010

**Published:** 2025-01-30

**Authors:** B. O’Mara, M. S. Mahmud

**Affiliations:** Remote Sensing Laboratory, Department of Electrical and Computer Engineering, University of New Hampshire, Durham, NH, United States

**Keywords:** rock climbing, bouldering, route grade difficulty, deep learning, machine learning

## Abstract

The determination rock climbing route difficulty is notoriously subjective. While there is no official standard for determining the difficulty of a rock climbing route, various difficulty rating scales exist. But as the sport gains more popularity and prominence on the international stage at the Olympic Games, the need for standardized determination of route difficulty becomes more important. In commercial climbing gyms, consistency and accuracy in route production are crucial for success. Route setters often rely on personal judgment when determining route difficulty, but the success of commercial climbing gyms requires their objectivity in creating diverse, inclusive, and accurate routes. Machine and deep learning techniques have the potential to introduce a standardized form of route difficulty determination. This survey review categorizes machine and deep learning approaches taken, identifies the methods and algorithms used, reports their degree of success, and proposes areas of future work for determining route difficulty. The primary three approaches were from a route-centric, climber-centric, or path finding and path generation context. Of these, the most optimal methods used natural language processing or recurrent neural network algorithms. From these methods, it is argued that the objective difficulty of a rock climbing route has been best determined by route-centric, natural-language-like approaches.

## 1 Introduction

Rock climbing’s popularity as a recreational sport is growing dramatically. It is a unique activity for both the body and the mind. In a puzzle-solving manner, climbers strategically scale vertical natural or artificial rock routes–a series of rock features–using their hands and feet. People are drawn to rock climbing because it is an activity in which one can improve physical fitness, problem-solving skills, and self-confidence (1, 2). It is estimated that the rock climbing gym market size was valued at 3 billion USD in 2023 (1), and this projected to double by 2032 (1). In the last five years, the establishment of rock climbing gyms in the US has grown by 6.46% per year (3). Within this time frame, three of the disciplines of rock climbing: sport climbing, bouldering, and speed climbing made their debut appearance at the 2020 Tokyo Olympics. More recently, it reached global audiences again at the 2024 Paris Olympics. Of the three disciplines, bouldering has the largest share of the rock climbing gym market (1, 2). This is because bouldering is the most accessible discipline of climbing, as it requires little equipment and technical knowledge. Recent trends in gym establishment highlight the increasing accessibility to bouldering. In the past decade, 50% of the gyms established in the United States and Canada are only bouldering gyms (3). To capitalize on this popularity, accessibility is crucial for climbing gym success.

Climbing accessibility is highly dependent on route setters. Route setters produce climbing routes, the central service of a climbing gym. They are responsible for producing routes that are varied yet consistent in difficulty. Gyms vary their route difficulties to capture the largest audience possible (1), catering to a range of climber experience levels from novice to advanced. However, the grading scales used to rate climbing route difficulty are often subjective according to the region, the gym, and the setter of the route (2). General factors considered when determining route difficulty are rock hold types, the number of rock holds on a route, the distance between the rock holds, and the angle of ascent (3). Therefore, it seems that the positioning and sequencing of holds are critical to route difficulty. But holds may be positioned and sequenced in an almost infinite number of ways. Setting a route is like composing a song (4, 5); there are constraints that govern its composition, but the liberty to operate within those constraints is quite large. When operating within these constraints, a route can be developed in a multitude of ways. This wide variance of route generation is a challenge for generalizing route difficulty. without a large sample size, route setters introduce their own biases when determining route difficulty, which then inadvertently affects the climber (i.e., the customer).

## 2 Motivation

Route setters are in an awkward position. The act of setting routes is inherently *subjective*, but the success of a climb depends on the ability of the setter to *objectively* set routes. This is the *Grading Bias Problem*: the setter of a route introduces their biases when declaring a route’s difficulty.

Reporting the objective grade of a climbing route is critical in the climbing community and can be aided by machine and deep learning technology (5). Increasingly, machine learning and deep learning techniques are being used to objectively classify the route difficulty. The objectives of this review article are to (1) understand how today’s route setters maintain objectivity in their setting, (2) to review the state-of-the-art approaches in determining climbing route difficulty with machine learning and deep learning, and (3) to suggest new areas for research. Together, these objectives are intended to address how climbing gyms can integrate machine and deep learning systems to streamline route setting and eliminate route difficulty bias for greater consistency and accessibility.

## 3 Document layout

The Grading Bias Problem will be thoroughly explored in subsequent sections of this paper. Section 4 provides context on rock climbing grade scales and how route setters currently set routes with the goals of objectivity and accessibility. Section 5 details the survey methodology and inclusion criteria. Section 6 identifies the approaches and methods of various deep and machine learning techniques to determine the climbing route difficulty and their success rates. Section 7 discusses the trends, performance, and shortcomings of current machine learning and deep learning techniques. In addition, it is argued that a route-centric approach with a natural language-like model is most optimal. Section 7 continues by proposing future areas for research, in which some proposals are based on works auxiliary to the survey.

## 4 Background: route grading systems and setting

**Figure 1:**
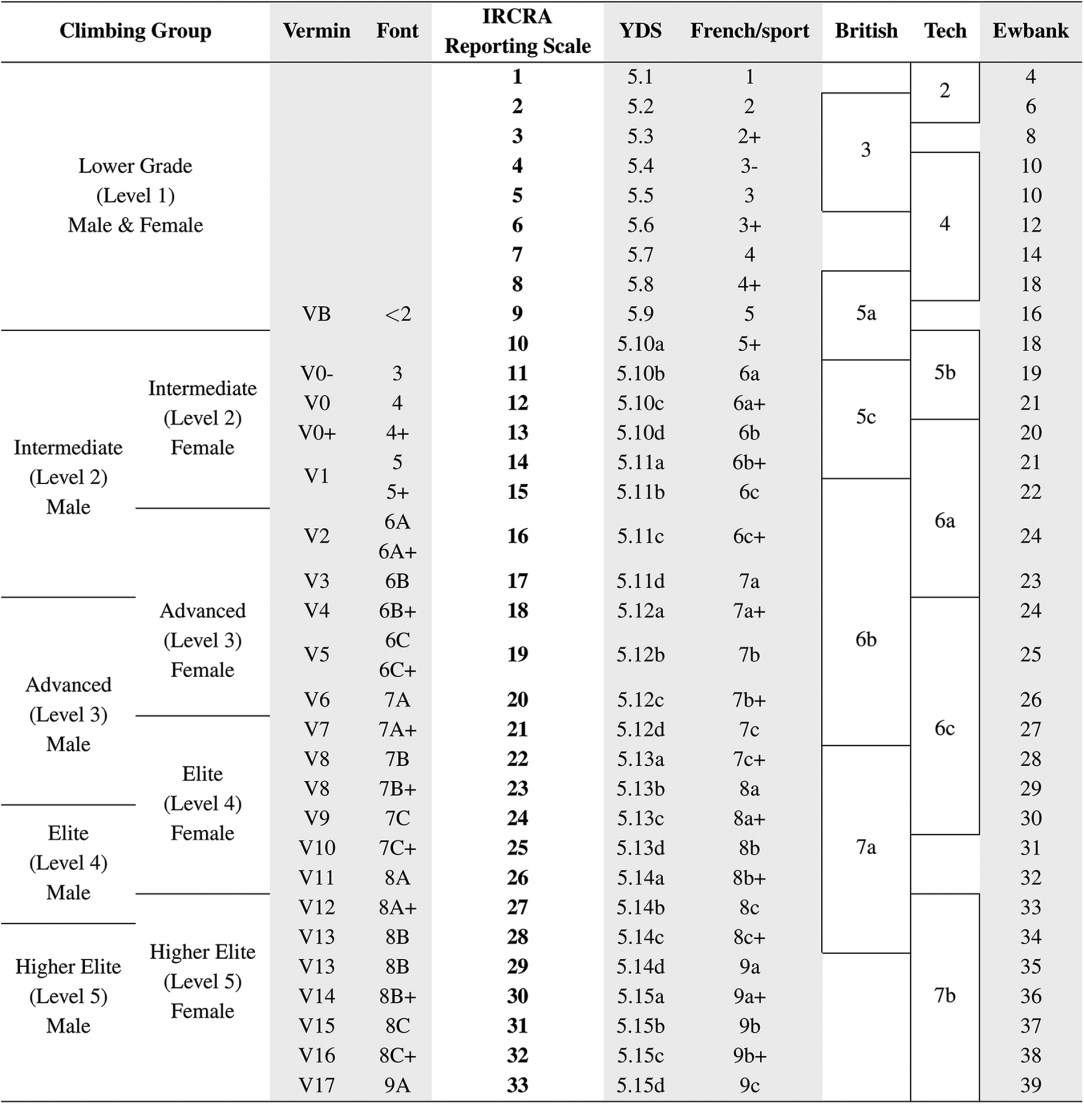
Climbing skill level across popular rock climbing route grading systems adapted from (5). The International Rock Climbing Research Association (IRCRA) established the IRCRA Reporting Scale for reporting the grade of a route as an integer value in rock climbing research. It may also be used as a reference for converting from one grading system to another. Note that the value of the IRCRA Reporting Scale has been increased by one since the first ascent of the V17 (9A) boulder route *Burden of Dreams*. Reprinted with permission under Copyright Clearance Center License Agreement. Boldface denotes the IRCRA Reporting Scale is a standardized scale recognized by rock climbing researchers internationally. Future researchers should consider reporting rock climbing route difficulties according to the IRCRA Reporting Scale to promote collaboration and ease of conversion to local/national climbing grades.

Climbing route difficulty can be graded on a variety of scales (Figure 1). The grade scale depends greatly on the discipline, subdisciplines, and climbing systems. In free climbing, the climber ascends a route without any artificial aid. The climber ascends a route by only the natural or artificial features of the rock. But a free climber can still use safety equipment (e.g., rope) in the event that they fall. In the subdisciplines of traditional (trad), sport, and ice climbing, of which the climber ascends a vertical face that is typically greater than 4 meters, the climber’s main tool for protection is a belay system consisting of rope, harness, and either temporary or permanent anchor points. The grading scales of these three “roped” disciplines account for *risk* to the climber in addition to the *technical difficulty* of climbing movement. Risk to the climber is most apparent in trad and ice climbing. In both trad and ice climbing, the climber sets and removes protection in rock crevices as they ascend. This protective gear is more prone to fail because they are not intended to be permanent fixtures in the rock or ice face. For ice climbing, it is particularly critical to gauge risk to the climber because the conditions of ice is greatly dependent of weather factors such as temperature, humidity, and precipitation. Although risk to the climber is still a concern in sport climbing, it is greatly reduced because protection gear is permanently fixed into the rock face. Furthermore, this predetermined protective gear reduces the cognitive strain on the climber because it outlines the intended route for the climber. How the risk and the technical difficulty of a route varies between grading systems.

Grading systems use is predominantly based on region. From the United Kingdom, the English Adjectival and Technical System is most notable for incorporating risk and technical difficulty. It uses a combination of a risk adjective (e.g., Easy) and a technical difficulty number (ranging from 4a,4b,4c,5a,…,7c) to classify route difficulty (8). For example, a moderate-risk, technically demanding route may have an adjective rating of M (Moderate) with a technical grade of 7a. This combines to a grade of M-7a. The use of a technical difficulty score developed as climbers increasingly ascended more difficult routes and the need for more granularity between difficulties increased.

A similar evolution occurred with the Yosemite Decimal System (YDS). The YDS scale is used in United States, and it ranges from 1.0 to 5.15d. The number in the one’s place denotes the class of incline and recommended equipment to minimize risk during ascent. Class 1 indicates walking on an even plane where no additional equipment is needed (8). Class 5 indicates a vertical wall ascent where the use of rope and other protection equipment is strongly encouraged to avoid severe injury or death in the event of a fall (8). Appending a decimal grade only occurs with Class 5. This decimal, ranging from 0.1-0.15d, further indicates the technical difficulty to ascend the route. More specifically, this decimal usually represents the difficulty of the most challenging part of the route. Like the English Adjectival and Technical System, the need for greater technical granularity increased as climbers kept ascending harder routes.

The French Sport System is widely used used in Europe. This system’s originates from mountaineers of the Alps (8). It, ranging from 1a to 9c (easiest to hardest) characterizes the overall technical difficulty of a route (8). This is unlike the English Adjectival and Technical, and the YDS scales because it (1) accounts mainly the technical difficulty of a route, and it (2) accounts for the overall difficulty of a pitch. A route is a set sequence of pitches. A route could be as short as one pitch, which is often defined by a rope length.

A newer scale used in Australia, New Zealand, and South Africa (9) is the Ewbank Scale. Its development stems from the English Adjectival and Technical System. This scale sought to simplify the characterization of route difficulty by assigning integer difficulty values starting at 1. The greatest numeric difficulty rating on this scale is currently 39; but, the intention of assigning integer difficulty values is so that the scale can continiuously evolve as climbers ascend harder routes.

**Figure 2:**
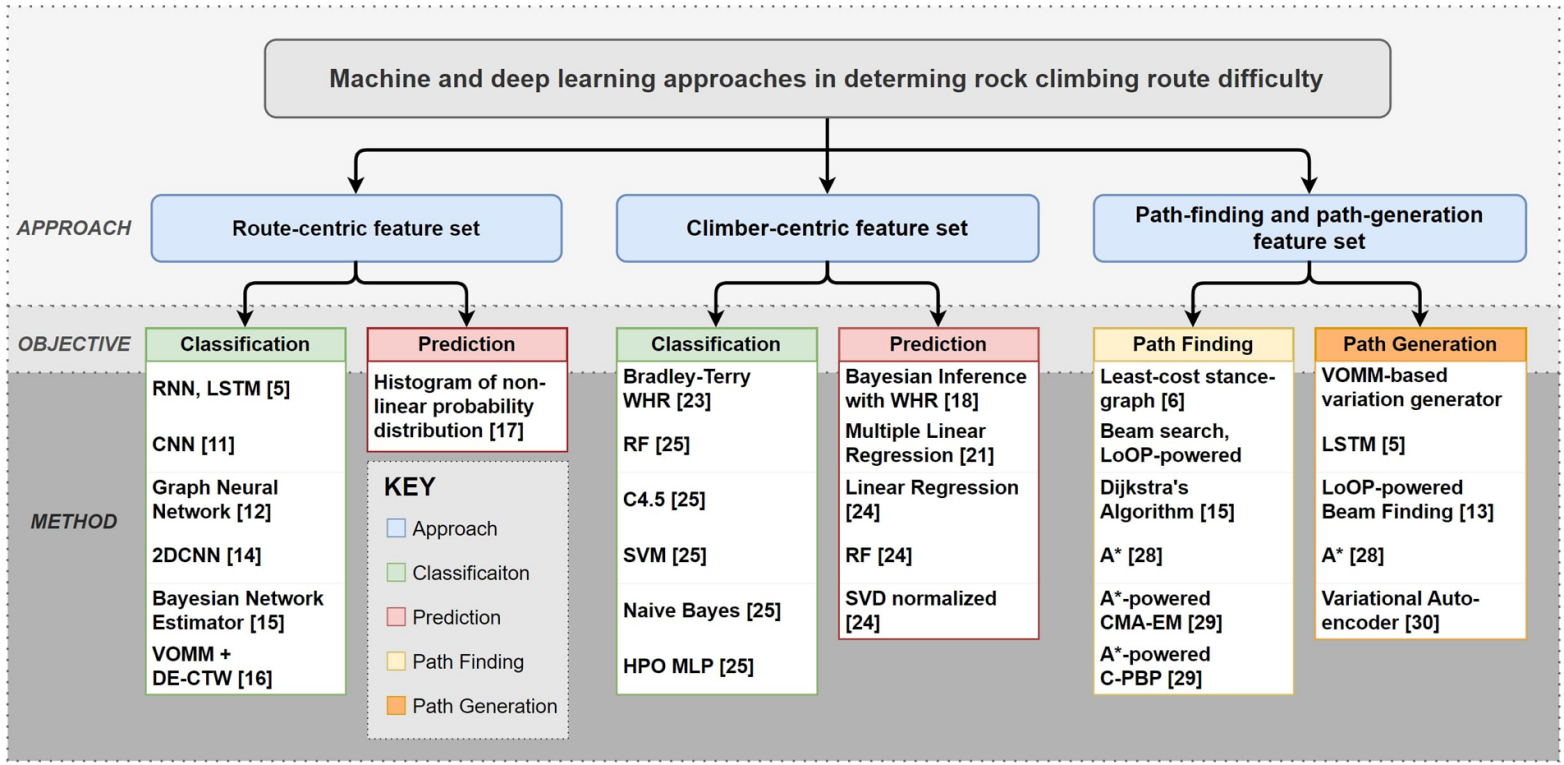
A categorical structure of approaches, objectives, and methods in determining rock climbing route difficulty.

Bouldering is not a “roped” discipline. Climbers ascend faces that are typically less than 4 meters in height. Protection usually comes in the form of foam padding at the base of the climb. When determining route difficulty, a key distinguishment between trad/sport and bouldering is that the bouldering grade systems only account for the *technical difficulty* of the climbing movement. The Vermin “V-Scale” is used in the United States and North America. This scale ranges from VB/V0 (easiest) to V16 (hardest). Plus and minus superscripts are sometimes appended to the V-rating to provide more granularity. In Europe, the most common bouldering scale is the Fontainebleau “Font-Scale” system. Similarly to the French Sport System, the Font Scale ranges from 1A to 9C; however, these are not one-to-one translations of route difficulty between the two systems. For example, a difficult beginner route on the Font-Scale, 6A, would translate to a French Sport System 6c+. This Font-Scale difficulty, 6A, may also be translated to the V-Scale, V3 (Figure 2).

Although bouldering grading systems are based on technical movement skill, route setters often take into account risk, intensity, and complexity (RIC). The RIC scale (10) is employed to ensure both variety and consistency at a specific route difficulty according to any bouldering grading system, and it serves to quantify risk to a climber. Each RIC metric is measured on an integer scale from 1–5 (Table 1). An RIC score for a route is then determined by taking the average of these metrics. This allows two (or more) routes of the same difficulty level to vary in risk, intensity, and complexity. So, the RIC scale may be used to generalize route characteristics across grading scales, and it may be used to identify differences between routes of the same difficulty grade.

**Table 1:** The Risk-Intensity-Complexity “RIC” scale used by route setters to ensure variety and consistency in route-setting (10).

Route description	Risk	Intensity	Complexity	Average
Crimpy, slab face demanding balance and forethought	3	2	5	3.33
Straight, over-hang vertical ascent with strong pinches	2	4	1	2.67

The first metric, Risk, gauges mental focus and commitment in a movement (10). This relates to how “scary” or how unsafe a move may feel to a climber (10); these moves can feel “low percentage,” meaning that the climber may not successfully make the move often. Intensity gauges the physical strength required to achieve the move (10). Finally, complexity gauges the technique and bodily awareness required to achieve a move (10). Technique differs from strength such that technique is dependent on the ability to shift and balance body weight, whereas strength is dependent on the muscles’ and tendons’ ability to execute a move.^1^ With these three metrics, let’s compare two V3 routes (Table 1). Route 1 has an R-I-C of 3-2-5, where its average RIC score is 3.33. Route 2 has an R-I-C of 2-4-1, a 2.67 RIC average score. Route 1 demands more problem solving and mental commitment from the climber, whereas Route 2 demands more physical strength. Overall, Route 1 may be a more difficult V3 than Route 2 because its RIC score is higher. The RIC scale allows route planners to create a variety of different routes at the same bouldering grade difficulty.

Route setters have an immense amount of responsibility. They have control over routes, the primary service of a climbing gym. They create routes for an intended climbing community, which can vary widely. To be a successful gym, its setters must be aware of their climbing community–the customer–and their demands (4, 11, 12). Mostly, setters should focus on producing routes that are accessible to 80% of their community (11). “Primarily…climbs should be equitable to all sizes of climbers in a given category” (11). This becomes especially important in competitions, such as the Olympics. How can objectively varied routes be fairly provided to athletes of varying physical abilities? What should be the measure of success in this personal sport, in which the true competitor is the climbing route itself?

Given the subjectivity and variety between climbing routes of the same difficulty between climbers, it is clear that the route difficulty could be customized to the climber. But the community adheres to standardized grading systems that attempt to objectively define climbing route difficulty.

In most cases, the difficulty of a climbing route is decided by whomever accomplished the first ascent. This is a well-respected practice among the climbing community; however, the opinion of a single climber or a few will inherently have bias. This bias leads to differences in perceptions of climbing difficulty between regions, gyms, and climbers. To mitigate this bias, some gyms have implemented ways for the community to input their ideas. Route setters will set for an intended difficulty grade (e.g., V3). Then they either (1) leave routes unrated until collecting difficulty grading input from climbers, or (2) rate a route and receive input from climbers. With this approach, the determination of route difficulty is based on a distribution, which opens opportunities for statistical and empirical substantiation. These approaches have lead researchers to use machine and deep learning techniques in determining rock climbing route difficulty.

## 5 Survey methodology

### 5.1 Search focus

The essence of rock climbing difficulty is multifaceted. Route difficulty is dependent on numerous factors pertaining to both the climbing environment and the climber. This survey investigates how these factors may be quantified with machine and deep learning techniques to provide a more objective determination of rock climbing route difficulty. The remainder of this section presents the conduct of this survey in detailing the inclusion and exclusion criteria, databases accessed, search queries asked, and identification of relevant works.

### 5.2 Inclusion and exclusion criteria

The base criterion to be included in this survey is that the study attempted to quantify the difficulty of a rock climbing route. These quantified factors may be derived from qualitative or quantitative measures. For example, a qualitative measure would be a climber’s perceived difficulty of a route while an quantitative measure would be the sequence of holds in a route. A study was included if route difficulty was indirectly quantified for an alternative end goal, such as finding an optimal route path or generating a new route. Said study would also need to explain the methodology of their route difficulty quantization.

Understanding indicators of climber performance is a burgeoning field of study within sports science. As the sport becomes more popular, researchers are seeking to formulate methods for improving climber skill. Although this survey focuses on the technology used in determining climbing route difficulty, references are made to climbing sports science to provide context for definitions of rock climbing difficulty, indicators of climbing performance, and how the sensing technology used to quantify climber performance can also be used to quantify climbing route difficulty. Overall, many works within climbing sports science were excluded because they did not directly attempt to quantify climbing route difficulty.

### 5.3 Search query

Eight databases were accessed: arXiv, IEEE Xplore, ACM DL, Semantic Scholar, Engineering Village, ScienceDirect, EBSCO Host. These databases were selected because they host articles pertaining to machine and deep learning and climbing sports science. Keywords and phrases searched in article document titles, abstracts, and bodies were: “rock climbing,” “grade,” “route difficulty,” “bouldering,” “classification,” “machine learning,” “deep learning,” and “bias.” Abstracts of resulting articles were read and then filtered if its content met the inclusionary criteria. The references section of those articles which met the inclusionary criteria were also reviewed. Twenty-two articles in total met the inclusion criteria.

These queries ended on July 2nd, 2024.

## 6 Machine and deep learning approaches, objectives, and methods in determining climbing route difficulty

It is critical to understand the different route grading systems when aiming to determine climbing route difficulty with machine and deep learning methods. Grading systems consider different factors when assigning a route grade. The YDS scale considers factors affecting both risk to the climber and necessary technical skill while the V-Scale only considers factors affecting technical skill. A YDS route dataset may then include feature data such as temperature, humidity, and available protection gear to account for risk to the climber, but this feature data would not be useful for a V-Scale route dataset. So, the performance of a machine and or deep learning model within one grading system may not be generalizable to another. If it a model were to be generalizable across grading systems, the grading systems must consider the same or similar factors in determining route grade.

The use of the words “determining” and “difficulty” are intentional in the phrase of “determining climbing route difficulty.” Both words are broad generalizations that capture the overarching goal of the work reported in this survey: to objectively quantify the amount of challenge a climbing route poses to a climber. “Difficulty” of a climbing route is an abstraction of its grade. Because there are various grading systems and granularity of challenge (i.e., the number of grades within a grading system), “difficulty” is used to describe the general challenge of a route. “Determining” is an abstraction of the outcome. Methods reported were aimed at either predicting or classifying climbing route difficulty, or generating climbing paths or climbing routes at a specified difficulty. In this survey, these aims of determining climbing route difficulty are categorized into a hierarchy of approaches, objectives, and methods.

• ***Approach**: a general description of the data features used as contextual information for determining difficulty of a climbing route.*
• ***Objective**: the resulting output of the model(s) and or algorithm(s) (i.e., prediction, classification, or generation).*
• ***Method**: the model(s) and or algorithm(s) used to determine difficulty of a climbing route in the context of an approach.*

Traditionally, the top hierarchical category for defining machine and deep learning models is by their objective, such as prediction or classification. But in this review, it was more appropriate to categorize works at the highest level by their approach; for, the feature data of one model may be used for prediction, whereas similar feature data of another would be used for classification. Three main approaches were identified: route-centric, climber-centric, and path finding or generation, where the latter is often a hybrid of the former two.

• ***Route-centric**: an approach whose feature set is dependent on the qualities of the route (e.g., hold types).*
• ***Climber-centric**: an approach whose feature set is dependent on the qualities of the climber (e.g., highest grade climbed).*
• ***Path Finding/Generation**: an approach whose feature set is dependent on both the qualities of the route and the movement of a climber.*

With these definitions, the methods were appropriately categorized. Figure 2 shows the organization of machine and deep learning methods in determining the difficulty of the rock climbing route according to the approach and objective. Flowing from the categories identified in Figure 2, Sections 6.1, 6.2, and 6.3 compare the methods for each approach in terms of their similarities, differences, and efficacy.

## 7 Discussion

### 7.1 The route-centric approach

The majority of route-centric models utilized features engineered from the MoonBoard route database. Its database contains over 30,000 bouldering routes created by users and MoonBoard itself. MoonBoard “is a standardized interactive training wall that connects a global community of climbers through shared problems and competitive performance rankings” (13). Users of the MoonBoard can upload bouldering routes and their difficulty grade on the standardized rock climbing wall of 198 possible holds^2^ arranged on a 18×11 grid. With each grid index denoting a rock hold, routes with an associated difficulty label are subsets of the 198 holds. In addition to providing route sequence data, MoonBoard also provides standardized images of a route on its app. Each image denotes the starting, intermediate, and end holds. Given that rock climbing gyms routinely set new routes by changing the holds themselves, the standardization of the MoonBoard and its route database size makes it a good launching point for machine and deep learning applications. The researchers (7, 14–18) used this launch pad to determine the difficulty of the rock climbing route with a route-centric approach.

Methods utilizing MoonBoard route data are among algorithms commonly used within the natural language processing (NLP) domain. If a route is defined by a set of holds, then a solution to the climbing route may be defined by the sequence of the holds and or the movement sequence of the climber about the holds. Phillips, Becker, and Bradley (6) laid the foundation for using NLP algorithms in describing climbing routes as a sequence of holds/movements with their route variation generator, Strange Beta. In their Climbing Route Descriptive Language (CRDL), they define how beta (instructions on climbing a route) may be parsed, sequenced, and given a difficulty rating. Their work is further discussed in the path finding and Route Generation Section. But it is necessary to indicate that their work was the primary inspiration for the ensuing NLP, route-centric approaches.

**Figure 3:**
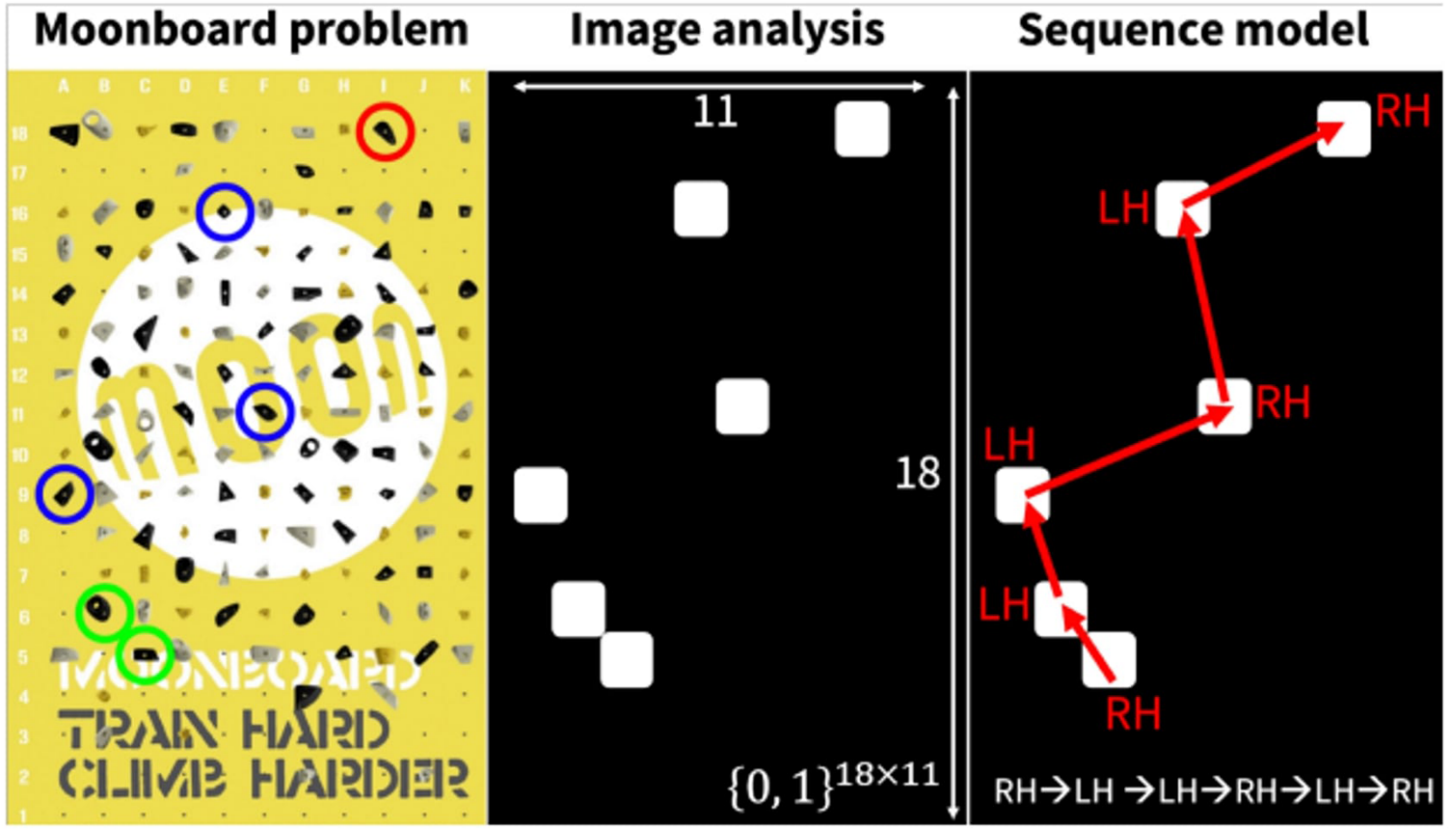
Duh and Chung’s (7) route pre-processor “BetaMove.” Reprinted with permission under CC BY-NC-SA 4.0.

Dobles, Sarmiento, and Satterthwaite (14) were the first to attempt classification of bouldering grades with MoonBoard 2016 route data. Of the three methods tested, their ordinal regression Convolutional Neural Network (CNN) classifier had the best performance. Although its classification accuracy, 34.0% across 13 different grades, was slightly less than the Naive Bayes and Softmax Regression models, the CNN generalized better to the distribution of route difficulty. There is a large class imbalance in the MoonBoard dataset, as it is skewed toward easier routes. The use of MoonBoard route image data was improved upon by Duh and Chang (7) with a Long Short-Term Memory (LSTM) Recurrent Neural Network (RNN). The key improvement in their approach was their pre-processor BetaMove (Figure 3), which sequenced route holds before passing through their grade classifier, GradeNet. GradeNet’s accuracy of 46.7% was an improvement from the CNN, but GradeNet was only classifying across ten different grades instead of 13. When allowed to have an error of ±1 grade, the classification improved to 84.7%, which was on par with human prediction accuracy.

While sequencing individual moves improved the accuracy, Petashvili and Rodda (17) demonstrate that similar performance can be achieved without this sequence pre-processing step. Instead, each route was one-hot encoded as a 18×11 feature vector (i.e., a route is a set of holds on the 18×11 grid; if present, that grid position is coded “1”). When passed through a 4-layer convolutional neural network (2DCNN) with a spatial-learning architecture, grade classification reached 42.0% accuracy across 12 grades, and it reached 84.0% accuracy when ±1 grade is permitted. Also similar to Dobles, Sarmiento, and Satterthwaite, Petashvili and Rodda found that their 2DCNN was less susceptible to class imbalances than classical regression models. This finding is significant for trying to improve the generalization of MoonBoard route difficulty prediction. Likewise, Tai, Wu, and Hinojosa (15) found generalization improvement with their using an graph convolutional network (GCN) over standard logistic regression. Their GCN, using one-hot encoded representations of routes, made neighbor comparisons to classify difficulty grade. Compared between neighbors were the route holds and grade. Some holds on the MoonBoard are associated with easier or harder grades; so, hold qualities can be key features for classifying route difficulty. With a Bayesian Network classifier, hold qualities such as incut depth features have achieved 71.0% accuracy across three difficulty grades (18). In continuation of building NLP-like models that classify climbing route difficulty, it is necessary to incorporate route-centric features concerning holds and movement between them.

The effectiveness of a machine and deep learning model is dependent on the quantity and quality of its dataset. MoonBoard’s crowd-sourcing approach makes its route database desirable for machine and deep learning applications. But crowd-sourcing data makes it difficult to control the quality of the data. Users introduce their own grading bias, and they may upload routes that clearly defy their claimed difficulty. But these undesirable affects on quality may be mitigated. To reduce grading bias, selection of routes should be limited to those whose difficulty has been determined by the community (7, 14). To reduce the affect of inaccurately graded routes, these routes should be removed (7). Then, when splitting the dataset into training and testing data, researchers can utilize the “benchmark” routes provided by MoonBoard. These benchmark routes are used as ground truth in (17) because they are uploaded by route setting professionals. While even professionals setters may introduce their own grading bias, it is they who possess some authority in determining the difficulty of a route.

**Figure 4:**
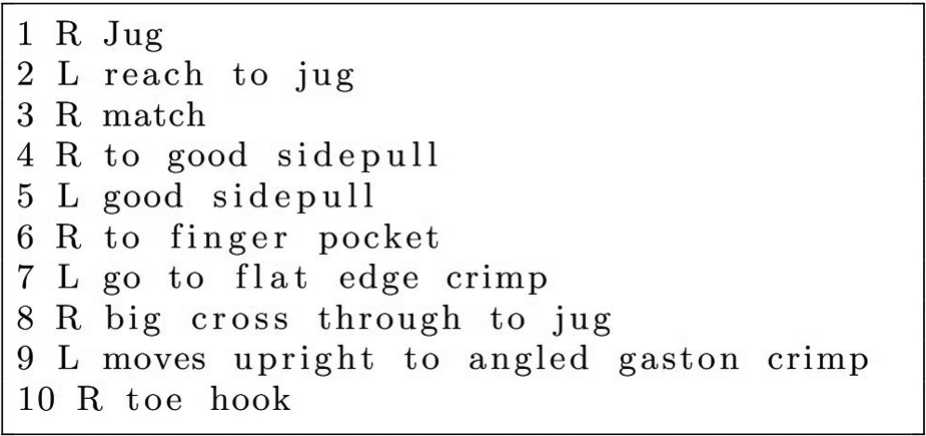
A route represented by a sequential instruction listing that is parsable and user-transcribed presented by Kempen (19) and adapted from Phillip’s et al. (6). A line describes one move by its sequence number, a left or right hand tag, and a free-form description. Reprinted with author permission from (19).

**Figure 5:**
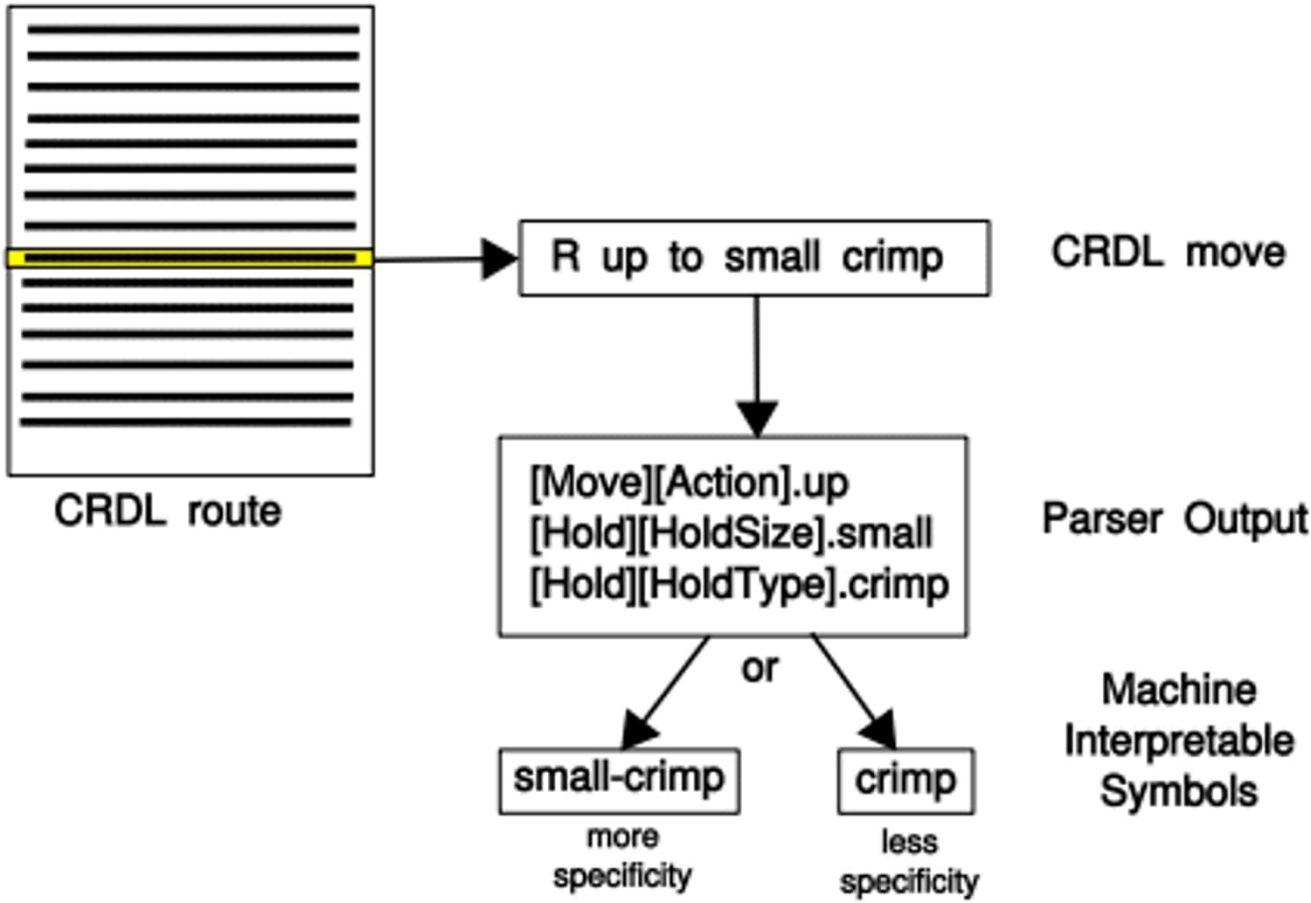
Strange Beta's CRDL parser of route beta into machine symbols presented by Phillips et al. (6). Reprinted from (6), with the permission of AIP Publishing.

The sequencing reminiscent of Strange Beta and BetaMove play a pivotal role in defining routes and their difficulty beyond the MoonBoard dataset. The sequence of hold and the movements between holds are intuitive features in an NLP solution within the route-centric approach. Climbers will often give advice on how to “solve” a route “problem” by giving sequenced movement instructions. Instructions or a set of instructions is called “beta.” Beta will be composed of two, sometimes three elements: which limb to move, which holds to move the limb, and sometimes a descriptor of the type of move (Figure 4). Using Strange Beta’s Phoenix grammar structure and parser (Figure 5), Kempen (19) modified this route generator to be used as a route difficulty classifier. This is an intuitive strategy, because climbers can infer the difficulty of a route just from its beta description. CRDL utilizes these colloquial descriptions of climbers on hold types, hold sizes, movements, and movement distances to classify the difficulty of the route. However, Kempen’s decomposed context tree weighting (CE-CTW) variable-ordered Markov Model (VOMM) only had 64.38% classification accuracy between two difficulties: easy or hard. It is the later emergence of a nonlinear probability distribution route grade prediction function that demonstrated accurate and granular performance on sequence-based, route-centric feature data.

Hold sequences demand effort from the climber. In between long sequences, some holds afford the climber the ability to rest and regain energy. Taking inspiration from the online classification calculator, DARTH GRADER,^3^ Ansel (20) built a probability-based model to predict the grade of the route. This model is based on the assertion that the perceived climbing difficulty of a route is non-linear (21); expounding on this relationship, Equation 1 states that there is a nonlinear relationship between the grade of the route and the energy expended by climbing to ascend the route on the following “energy of reference” (20):

(1) $$E=1.21^{2n}$$

where n is the integer translation of a route grade (20). The integer value for route grade, n, can be determined by the individual route sequences and quality of rests as defined by Equation 2:

(2) $$g_{n}Rg_{k}$$

where $g_{n}$ is the grade of one sequence, $g_{k}$ is the grade of the following sequence, and R is the quality of the rest in between the sequences (20).

**Figure 6:**
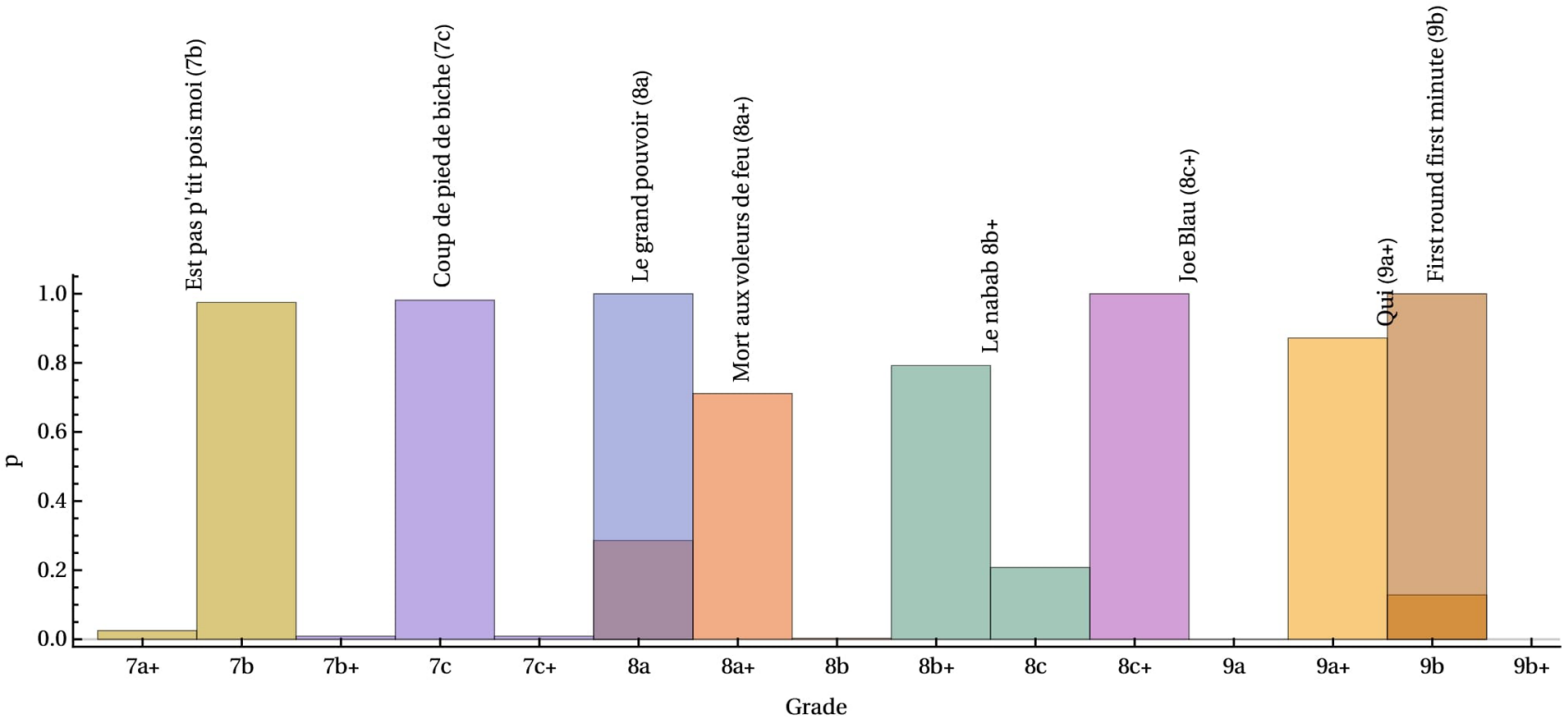
Probability of route grade $p(g_{n})$ predicted by the model presented by Ansel (20). The route names indicate the official route grade whereas the histograms indicate the probability of each respective route grade. Reprinted with permission under CC BY 4.0.

This method had great success in predicting the probability of official sample grades. The predictions of the grades were highly concentrated on their official grade, as seen in Figure 6. Furthermore, a meta-analysis of the model’s classification accuracy demonstrates that the model has a high accuracy of 91.75%. Although the sample size of the meta-analysis was small, these results suggest that hold and movement sequence are critical to classifying or predicting route difficulty using a route-centric approach.

The route-centric approach refers to feature data of the route. Common route features to consider were holds, hold types, movements between holds, good and bad qualities of holds and movements, and graded sections of route. But the most important factor in producing high performing models was to sequence this feature data. This is an intuitive method because this is how human climbers would assess the difficulty of the route. Climbers often preview routes (22) to break down their structure (holds), required movements, and the sequencing of movements between hold positions. As climbers do in previewing, researchers should explore computer vision techniques to extract specific route-centric features. Then, these may be fed through an NLP model to classify a route’s difficulty.

For further comparison between route-centric methods, see the summary of each method in Table 2. It highlights key model information, such as feature set, granularity, and performance.

**Table 2:** Route-centric machine and deep learning methods used to determine rock climbing route difficulty.

Source	Objective	Method(s)	Feature data	Route difficulty granularity	Metric of success	Performance
(19)	Classify route as easy or hard	VOMM, DE-CTW	Enriched CRDL	2 (Easy/Hard)	Classification Accuracy	64.38%
(7)	Classify route difficulty	RNN, **LSTM**	Hold sequence data from MoonBoard 2016 routes	13 (V4-V13, V-Scale), **13 (V4-V13, V-Scale±1)**	Classification Accuracy	46.7%, **84.7%**
(17)	Classify route difficulty	2DCNN	Hold sequence data from 2016, 2017, 2019 MoonBoard routes	21 ( Fontainebleau), **21 (Fontainebleau±1)**	Classification Accuracy	42.0%, **84.0%**
(16)	Classify route difficulty	LoOP-powered HGBC	Scored hold sequence data from MoonBoard routes	11 (V4-V14, V-scale)	Classification Accuracy	46.5%
(14)	Classify route difficulty	CNN	2016 MoonBoad route data	13 ( Fontainebleau Scale)	Classification Accuracy	31.8%
(20)	Classify route difficulty	Histogram of custom non-linear probability distribution function	Energy needed to complete a route section, rest quality in between sections	21 ( Fontainebleau scale)	Classification Accuracy	91.75%^a^
(15)	Classify route difficulty difficulty classification	Graph Neural Network	MoonBoard 2016 routes	11 (V4-V14, V-Scale)	AUC	0.73
(18)	Classify route difficulty	Bayesian Network Estimator	MoonBoard 2016 route data (number of holds, distance between holds, types of holds, and incut sizes of holds)	3 (partial Fontainebleau)	Classification Accuracy	71.0%

Bolded terms indicate the best performing model when authors observed the performance of two or more methods.

^a^ This accuracy was calculated via a meta analysis of Figure 3 of (20).

### 7.2 The climber-centric approach

It may seem that shifting the focus from the route to the climber is a move toward greater subjectivity. However, the characteristics of climber performance and the performance history are indicators of the difficulty of the route.

“It can be said with certainty that an evaluation of the difficulties, in any part of the world be defined, is used to quantify the ’performance’ that a mountaineer or climber must express in order to overcome a wall, a step, a block” (8).

Two main themes emerge in the climbing-centric features. The first is the use of wearable sensors and or recording climber bio-metrics. These metrics, such as electromyography (EMG) (23) and acceleration (24), gauge the physical performance necessary to ascend a route. The second theme departs from the first in that the utilized features are from past performances of the climber. These past performances, often recorded in a logbook (21, 25, 26), can help infer the rating of the difficulty of the route. But for both themes, portable smart devices play an important role in collecting data. As with activities such as running, cycling, and swimming, fitness products and apps may soon be instrumental in tracking climbing activity and skill progression. Furthermore, they may be instrumental in determining rock climbning route difficulty through a climber-centric approach.

Although understanding of high-skill climbing performance indicators is an evolving sport science, that knowledge base has given insight to determining route difficulty from a climber perspective. To base route difficulty on climber skill, the assumption is made that climber skill performance degrades at difficulties beyond the climber expertise. Ebert (27) observed the following indicators of skill degradation: inaccurate gripping, increased use of strength during transition periods, trembling during rest periods. Such metrics indicate the degradation of the “core” climbing abilities: power (transfer of isometric strength into a move), control (smooth transitions between holds), stability (maintained composure), and speed (rate of ascent) (28). In automatically classifying climbing route difficulty, Ebert (27) hypothesized that as the difficulty of a route increases, so does the difficulty in maintaining the four aforementioned climber abilities. Across 153 ascents, 13 different bouldering routes, and 3 different difficulty ratings, human acceleration and rotation data collected from the limbs and chest were collected and feature engineered. Of the five models tested, it was the hyper parameter optimized (HPO) multi-layer perceptron (MLP) that produced the best classification accuracy of 98.04%. Interestingly, this accuracy measure was reached by ignoring the acceleration and rotation data captured by the chest. Other than acceleration and rotational data, EMG activity of the forearm (more specifically, the flexor digitorum profundis) was shown to have a logarithmic relationship in predicting route grade (23). As observed in (21), an objective measure of route grade becomes more difficult to distinguish at higher grades.^4^

In addition to bio-metric tracking, another desirable tracking service in climbing concerns route ascent history. Researchers (21, 25, 26) have scrapped databases from logbook apps like theCrag and Vertical-Life to determine climbing route difficulty. Route ascent history has shown a unique relationship between climber skill and route grade in rating system algorithms. Whole history rating (WHR) methods posit the climber and the route as two-players in an adversarial, Bradley-Terry game of odds. The game has one of two results: the climber “sends” (ascends) or fails to ascend the route. There are two key independent variables at play in this game. The first is the time-varying climbing grade of the climber (21, 25, 26). In instances when the climber’s climbing grade is self-reported, it is still a valid measure of the climber ability (29). The second is the grade of the route. Equation 3 describes the probability of sending a route as a function of the route grade and the time-varying climber grade.

(3) $$p_{\mathrm{send}}=\frac{e^{mC(t)}}{e^{mC(t)}+e^{mR}}$$

where $p_{\mathrm{send}}$ is the probability of sending a route of grade R for a climber of grade C(t) optimized for some parameter m.

This method of route difficulty determination was first presented by Scarff (25) and later improved by Drummond and Popinga (21). The latter improved by describing the increase of route difficulty through the application of a Bayesian Markov Chain Monte Carlo inference on the timing-varying climber skill grade. Although Drummond and Popinga used a different method than Delignieres et al. (23), they observed a non-linear model for route difficulty. For example, on the Vermin scale, an increase in grade corresponds to a 3.17 increase in difficulty (21). This demonstrates that despite the feature data, perceived increases in difficulty do not follow a linear scale; and logbook data has the potential to better describe this non-linear relationship in climbing route difficulty.

**Figure 7:**
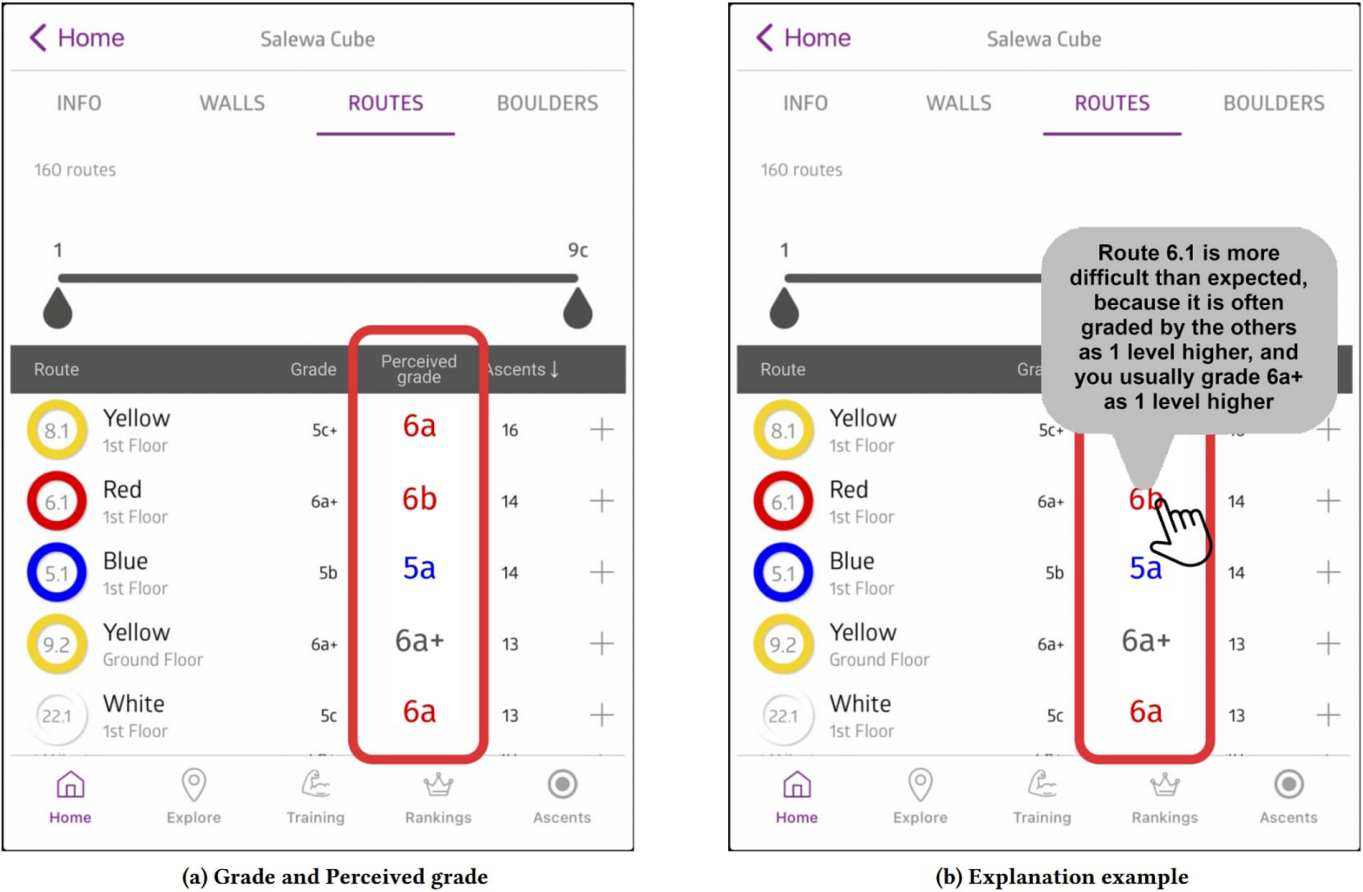
An example of interface for browsing indoor climbing routes presented by Andric et al. (26). Figure **(a)** shows how a climber can observe route setters' grades and his/her predicted perceived route grades in columns 'Grade' and 'Perceived grade', respectively. Red colour of a perceived grade indicates that the route would be perceived as more difficult, while blue colour indicates that the route would be perceived as easier than the official route grade. Figure **(b)** illustrates an example of automatically generated explanation for the predicted climber's perceived grade of a route. Reprinted with author permission from (26).

A limitation in logbook data is that the data is reliant on the self-reporting of climbers. Climbers may be more apt to log ascents than failed attempts (21), and exploration of their logging biases and behaviors (21, 25) is nascent. There are multiple ways to mitigate this bias. One would be to offer a tool that automatically captures sends, attempts, and failures. For example, the ClimbSense inertial measurement unit bands (24) could accurately recognize when a climber sent a route. This activity recognition accuracy hardly degraded when only one band was used, suggesting that climbing activity recognition may be achieves with smartwatches and other wrist-worn fitness gadgets. Another mitigation technique is to learn climber bias. Andric et al. (26) focused on understanding the perceived difficulty of a route in their climbing recommendation system. As seen in Figure 7, they propose how this recommendation system would learn the bias of a climber and make suggestions based on their perceptions. An example statement (in reference to Figure 7) would be:

“You could perceive route r as more difficult than officially graded, because it is often graded by other climbers as 1 level higher, and you usually grade 6a+ as 1 level higher” (26).

Their knowledge-based models, operating with engineered features based on time-varying properties of route grade and climber grading, predicted perceived difficulty of indoor and outdoor routes. For the indoor routes, their Random Forest regression model performed 9.5% better than the baseline recommender system (26).

The determination of difficulty of the climber-centric route is based on performance and performance history. Common feature data to exploit were biometrics recorded from wearable electronics or from logbook databases. It is unclear which will become the most efficacious; however, it is clear that tracking climbing performance and performance history are burgeoning research fields that will be enabled by ubiquitous computing devices and cloud services. For further comparison between the climber-centric methods, refer to the summary of each in Table 3. It highlights key model information, such as feature set, granularity, and performance.

**Table 3:** Climber-centric machine and deep learning methods used determine rock climbing route difficulty.

Source	Objective	Method(s)	Feature data	Route difficulty granularity	Metric of success	Performance
(25)	Classify successful and unsuccessful ascents	Bradley-Terry WHR	theCrag outdoor climbing ascent outcomes and time-varying climber grade	2 (Fail/Success, Ewbank Scale)	Classification Accuracy	85% (average)
(27)	Classify route difficulty	RF, C4.5, SVM, Naive Byes, **HPO MLP**	3D Acceleration and rotation data of human limb movement and chest	3 (Partial Fontainebleau Scale)	Classification Accuracy	79.01%, 68.63%, 86.93%, 75.82%, **98.04%**
(21)	Predict route grade	Bayesian MCMC inference of Bradley-Terry WHR	theCrag outdoor climbing ascent outcomes and time-varying climber grade	N/A (Ewbank, Font, and V-grade Scales)	N/A	N/A
(26)	Predict climber’s perceived difficulty of climbing route for route suggestion	Linear Regression, **RF Regression**, SVD normalized	Static and time variant official and climber-rated route grades on the Vertical Life app	32 (Font Scale)	RMSE (Indoor Routes)	0.381, **0.378**, 0.384
(23)	Model route difficulty based on perceived difficulty	Multiple linear regression fitting perceived difficulty	Perceived exertion, perceived motor accuracy	16 (Font scale)	Correlation coefficient	r=0.993

Bolded terms indicate the best performing model when authors observed the performance of two or more methods.

### 7.3 The path finding and path generation approach

path finding and path generation incorporates both route-centric and climber-centric features. Both are dependent on the holds of the route and the sequenced movements of the climber. In the context of rock climbing, path generation is the equivalent of route setting, and finding a path is the climber equivalent of finding a “solution.” If a route is a set of holds, then a solution is the set of sequenced movements to ascend the route. Furthermore, the most optimal solution would be the one with the lowest path finding cost (9, 16, 18, 30, 31). These costs incorporate restrictions imposed by the route and the features of the climber movement in path finding and path generation.

**Figure 8:**
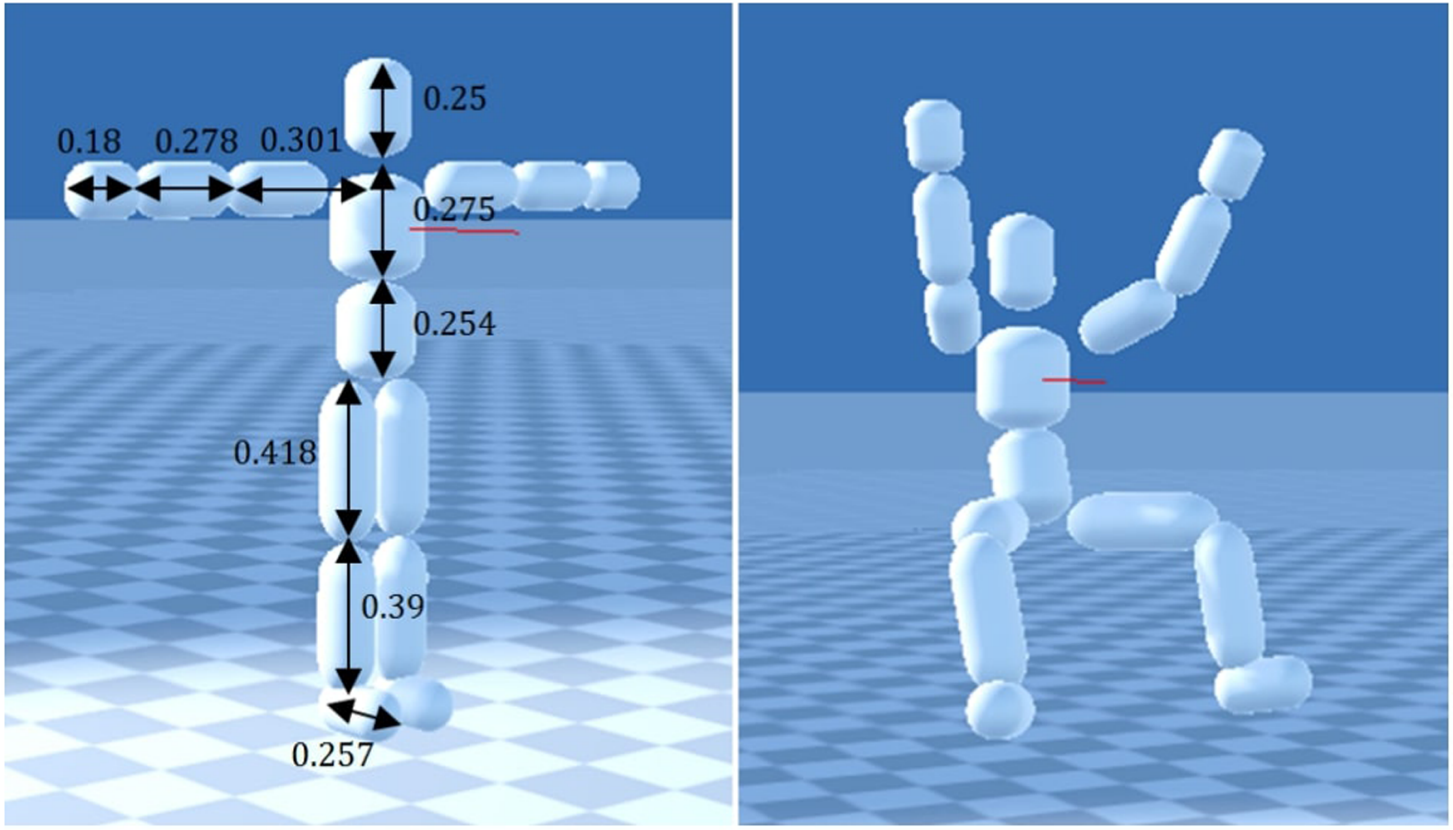
Simulated humanoid climber posture presented by Naderi et al. (31). Left: climber in T-pose with measures in meters. Right: Climber's preferred posture. Reprinted with permission under Copyright Clearance Center License Agreement.

The A* algorithm and its relatives have commonly been applied to finding the path of solution. These algorithms: beam search (16), least-cost stance graphs (9), Dijkstra’s shortest-path algorithm (18), and A* (31), are well suited to find the least-cost solution. A least-cost solution for the climber is the one that expends the least amount of energy. As seen earlier from Ansel (20), energy expenditure is primarily dependent on the route hold sequence and the movements required to move through the sequence. Although the costs for each algorithm varied between researchers, the costs focused on nodes and edges. The nodes–or stances (9)–represent the posture of the climber on a set of holds. Mathematically, climber posture can be defined by a 4 limb/hold hypernode (18) or a 4-tuple of holds (9). Edges represent the movement of a climber between nodes (postures). For node costs, emphasis was placed on positioning the climber agent in a preferred climber stance, which is depicted in Figure 8. The preferred climber stance is one that has unique hold for each limb, a low center of mass (COM), and often keeps feet below hands. For edge costs, emphasis was placed on mitigating dynamic movements. For both nodes and edges, costs were introduced to limit postures and movements to what is humanly possible. A comprehensive list of observed node and edge costs is further detailed in Table 4.

**Table 4:** Common costs for nodes (body posture defined by limb positions) and edges (movements between nodes).

Cost category	Cost	Reason for cost	Source(s)
Node	free limbs	It’s undesirable to have a free hand; somewhat undesirable to have a free foot	(9, 30, 31)
	unique limbs	Matching limbs on the same hold, especially feet, is often undesirable	(9, 30, 31)
	center of mass (COM)	It’s desirable to have COM low and near wall; undesirable to have limbs close together	(9, 30, 31)
	stretch/reach	It’s undesirable to be at maximum reach	(9, 16)
	matching feet	Matching feet is often undesirable for balancing weight	(9)
	limb crossing	Maintaining crossed limbs is difficult	(9, 30, 31)
	high feet	Unless in instances of heal-hooking or toe-hooking, it is undesirable to place feet high	(9, 30)
	hold type	Hold type influences the difficulty to maintain a posture	(9, 16, 30)
Edge	cumulative distance between all holds	Need to keep limb distances within human limits	(9, 31)
	limbs changing	It’s desirable to move one limb at a time as it may cause instability	(9, 31)
	optimal deviation from current node COM to next node COM	It’s not desirable to always make dynamic movements	(16, 31)
	flow (comparison of movements)	It’s desirable to have varying movements in a climb	(16)

Despite these costs, it is not clear how to evaluate an optimal solution or how to evaluate the difficulty of a climbing route. In fact, Turedioglu et al. (18) was the only cohort whose primary objective was to find the most optimal solution. They used MoonBoard 2016 route solutions as ground truth. In comparison, their solutions matched the MoonBoard nodes with accuracy 39% in three graded routes. Naderi et al. (31) was concerned with modeling climbing movement moreso than finding an optimal path. Their simulated climber agent could reflect either “skilled” or “hobbyist” climber movement. This suggests that if the climber agent were applied to a MoonBoard set, its movement pattern could adapt the difficulty grade of route. This would have the potential to infer the difficulty of a route based on climber movement. These climber movements were the basis for Stapel’s (16) route difficulty classifier. Stapel’s method was a four-step process: (1) use beam searching to find the optimal solution, (2) grade the difficulty of each movement in the solution, (3) sum these difficulties, and then (4) classify the difficulty of a route. This method is similar to the NLP method of Ansel (20), but the poorer classification accuracy–46.5% across 11 grades–of the HGBC suggests that the movement feature data was not suited for the problem or that a higher-level approach to sequence data is more appropriate. Celik (9) was concerned with classifying the difficulty of the route and the order of the difficulty of the route, respectively. As for Celik, his least-cost stance graph model, based on hold property feature data, had a 75% model fit in classifying route difficulty ordering. But this was only across five grades. The pathfinding approach in determining climbing route difficulty is in its nascent stage; but this approach, an approach that directly mimics sequenced climber movement, demonstrated some success, and it has potential to improve.

Pathfinding and path generation are closely related processes. With some modifications, Stapel’s (16) beam-searching method generated quality MoonBoard routes. His overall method was changed by personalizing costs to a climber with climbing action capabilities. For example, the stretch/reach cost (Figure 4) was customized to the actual ape-index of the participants (16). Incorporation of these climber-centric methods into route generation reportedly created MoonBoard routes that were enjoyable, had good flow, and were on par with the benchmark routes produced by MoonBoard (16). The climbing abilities that had a large effect in creating these quality routes were the level of difficulty, reach, power, and finger strength. So, in this case, the difficulty of a route is customized to the strengths or weaknesses to the climber, opening up possibilities for custom training and defining route difficulty from the climber perspective. To have a more route-centric, route generation approach, the difficulty of holds may be incorporated. Katsura et al. (30) investigated which degree of hold difficulty stratification would produce accurately graded routes with the A* algorithm. Across 4 grades, climbers rated the MoonBoard routes generated with eight degrees of hold difficulty stratification as the most accurate for the intended grade (30). The degree of hold difficulty granularity has a large effect on the quality of route generation. As pathfinding and pathgeneration are closely related, it is likely that the knowledge gained from route generation would be insightful to more accurately determine route difficulty based on pathfinding.

Not all route generation algorithms have path finding roots. Instead, many generate climbing routes through NLP methods. The aforementioned Strange Beta (6) (VOMM-based variation generator) was the first computational tool with the aim of generating climbing routes. Its main intent was to aid route setters in creating routes, which was met with apprehension. Two route setters were “hesitant to endorse anything that would lessen their creative control” (6), and they found the tool “tedious” (6) to use. Furthermore, Strange Beta was sometimes unwieldy and created “chaotic” (6) 5.10 routes absent of climbing flow. This, and generating routes that are unclimbable, has been a problem with other NLP-like route generation tools (7, 32). However, climbers did prefer the 5.11 routes made with the assistance of Strange Beta. A later variational autoencoder development was also met with mixed success in generating MoonBoard routes, where less than half of the generated routes were deemed climbable (32). But Duh and Chang’s (7) DeepRouteSet (RNN+LSTM) had greater success in generating MoonBoard routes, where 80% were deemed of high quality and 95% were deemed reasonable for the difficulty. Among the route generation approaches, the NLP method has proved the most successful.

There is no standard for evaluating the quality of a generated climbing route. In general, researchers (6, 7, 16, 30) have surveyed climbers and or route setters to assess the quality of the route. Likert-like qualification scales were commonly used as the evaluation method. Statements such as, “Route or movement is appropriate for the grade,” is a helpful qualifier in gaining insight on how well a generated route aligns with the target difficulty grade. Other useful qualifying statements and questions are detailed in Table 5.

**Table 5:** Examples of qualification statements and questions for evaluating a generated rock climbing route.

Qualifier statements and questions	Source
Route has good flow throughout the climb	(6, 16)
Route has high-quality, quality of a benchmark	(7, 16)
Route or movement is appropriate for the grade	(6, 30)
“Climbing the route was enjoyable”	(16)
“Route fits my climbing style”	(16)
Design of route promotes an aspect of climbing training	(16)
“Movement is close to expert climbers”	(30)
“I think this move is reasonable for the course and difficulty”	(30)
“Is the problem [route] decent/reasonable?”	(7)
“Difficulty is consistent throughout the climb”	(6)
“Appears to be well thought out”	(6)
“Climbs awkwardly”	(6)
“Is creative/has interesting moves”	(6)
“Crux is technically engaging”	(6)

Pathfinding and path generation methods to determine the difficulty of the rock climbing route combine qualities from route-centric and climber-centric approaches. Their feature set depend on both route properties, like hold type, and climber properties, like movement abilities. While many route generation methods use path finding algorithms, the more successful ones use NLP-like. The corpus of path finding and route generation methods related to determining the difficulty of a route or producing a route whose grade is suitable for the difficulty may be viewed in Table 6.

**Table 6:** Path-finding and path generation machine and deep learning methods used determine rock climbing route difficulty and or produce a route accurately at a difficulty grade.

Source	Objective	Method(s)	Feature data	Route difficulty granularity	Metric of success	Performance
(7)	Generate bouldering routes	LSTM	Hold sequence data from MoonBoard 2016 routes	13 (V4-V13, V-Scale), **13 (V4-V13, V-Scale±1)**	Climber qualification	95% routes were reasonable, 80% of high quality
(6)	Generate new or variant climbing routes	VOMM-based variation generator	A climbing route descriptive language	2 (5.10 and 5.11 on YDS Scale)	Likert-type scale-based Wilcoxon rank-sum qualification by climbers	No preference for 5.10 climbs, 5.11-generated climbs were preferred
(16)	Classify route grade	LoOP-powered beaming searching-informed HGBC	Scored hold sequence data from MoonBoard routes	11 (V4-V14, V-scale)	Classification Accuracy	46.5%
(16)	Generate bouldering routes	LoOP-powered beam searching	Climber action capabilities, stance and movement costs	11 (V4-V14, V-scale)	Likert-qualification of route quality by climbers	Enjoyable, quality, and flow were on par with benchmark routes; were deemed more unsafe
(9)	Classify order of route difficulty	Least-cost stance-graph path finding algorithm	Wall position, hold position, hold direction, hold type	5 (partial Fontainebleau)	Fitness function	75%
(30)	Generate climbing routes at specific difficulties	A* path finding	MoonBoard 2017 routes, hold type difficulty	4 (Dankyu & Font)	Likert-qualified accuracy by climbers	8-hold difficulty granularity was preferred
(18)	Find most optimal climbing path	Dijkstra’s Shortest Path Algorithm	MoondBoard 2016 route data broken into stances (hypernodes) and movements (hyperedges)	3 (partial Fontainebleau)	Accuracy comparison with MoonBoard suggestions	39.0%
(31)	Simulate climber movement and determine optimal climbing path	A*-powered CMA-ES, A*-powered C-PBP	Artificial climbing wall, 8x4 max	N/A	Researcher and climber qualification	Both produced plausible movements, CMA-ES produced skilled movement movement
(32)	Generate MoonBoard climbing routes	Variational Autoencoder	MoonBoard 2017 route holds	Unspecified	Researcher and climber qualification	22/50 routes were climbable

Bolded terms indicate model achieved it's best performance when allowing for a +/-1 error in generating a route at a specified grade.

### 7.4 What’s the optimal approach?

The main products of a rock climbing gym are routes. Route production quality is reliant on the route-setting team. They aim to provide routes of varying–yet consistent–ratings of difficulty for their community of climbing customers. A detriment to consistent route setting are the personal biases that route setters introduce. In attempting to address this grading bias, a machine learning and or deep learning model tool may be helpful in assisting route setters. This optimal tool would be able to *accurately* determine route difficulty with with great *granularity*. The accuracy and granularity of route grade determination are the key outputs to optimize.

Sequence is the key. Route-centric, NLP and probabilistic methods were the most successful in this definition of optimal outputs. On a standardized rock wall, Duh and Chang’s (7) RNN-LSTM GradeNet achieved the greatest granularity accuracy (84.7%) when allowed a ±1 deviation from the actual route grade. It was the MoonBoard hold data, when sequenced, that allowed for such a high accuracy. For chaotic (non-standardized) representations of rock walls, the probabilistic, HGBC model (20) achieved the best accuracy (91.75%) observed by any model. As with the RNN-LSTM architecture of GradeNet, the sequencing of feature data (expended climber energy and quality of rests) was profound in determining route grade for the HGBC. It is evident that successful determination of route difficulty is dependent on sequencing.

### 7.5 Future work

There are limitations to the identified optimal models. The first limitation being that GradeNet operates on a dataset from a standardized rock wall (i.e., MoonBoard). While MoonBoard’s standardization makes it desirable for machine and deep learning models, most frequently climbed routes at climbing gyms are not on a MoonBoard. Most frequently climbed routes at a climbing gym are non-standardized, varying widely in length and path. These routes are also time dependent. Gyms continually update their walls with new routes. This is a situation where a model like the probabilistic HGBC (20) would be more appropriate; for, it relies on graded sections of a route, not the individual holds and their geometries relative to each other. But the limitation of the probabilistic classifier is that it does rely on *pre-graded* sections of a route. These pre-graded sections may introduce a grading bias. To mitigate this from a route-centric approach, each pre-graded section would need to be evaluated by a large number of climbers, such as with the community-determined routes in the MoonBoard database. There is thus an opportunity to develop a route rating system for climbing gyms. Members of a gym community could grade and rate routes, effectively building a large grading database for the gym. Building this database already is the app Crux, which allows users to set their own routes, grade their own routes, and grade the routes posted by others. The path-finding approach would also be suitable for determining route difficulty on a non-standardized wall. Each section of a route could be broken down into individual hold and move components as in (16). This would circumvent the need for relying on crowd-sourced grading data. Instead, the onus would be on route setters to upload their routes complete with hold and sequence features to an online path-finding model for grade evaluation. But this may prove labor to be a labor intensive, which opens an opportunity for computer vision to help.

Computer vision would be helpful in route-centric and path finding and path generation approaches. Besides the image pre-processor GradeNet, computer vision has seldom been used in determining rock climbing route difficulty. Image pre-processing could perform feature extraction on standardized and non-standardized rock walls. Features of interest within the route-centric and path finding approaches would revolve around holds and climber movements. Hold type, size, placement, and distances between holds would be good features for breaking down the difficulty of hold sequences, which builds on the hold difficulty stratification of (30). Contributions in distinguishing holds from the wall have recently been made in both research (33) with Climb-o-Vision and apps like Crux. Moving forward, a standardized image database of rock holds would need to be established. In this database of rock holds, it would be important to label each image with hold type, size, quality of hold incut, angle of climber observation, and the angle of incline. Climber movements would also indicate difficulty. Some movements are harder to perform than others. For example, a heel-hook often requires less energy to perform than a Gaston. A heel-hook involves placing the heel of the foot on a hold to pull oneself toward the wall. This movement engages the leg muscles, which can take more strain than the arm muscles, thus allowing more conservation of energy. A Gaston, where the climber’s hands pry opposed with elbows flared outward, is a powerful movement that engages the shoulder and back muscles of the climber. If a computer vision pre-processor could accurately distinguish climber movements, it could then rate the difficulty of a movement and grade the difficulty of sequenced movements.

Recording human movement may also be approached in a climber-centric context. A Gaston is typically a strenuous movement to perform. However, it may not always be a hard movement. Wearable and embedded sensors have the opportunity to measure and quantify the exertion of the climber as an indicator of difficulty. On the wearable side, metrics of heart rate (34), EMG activity (23), and acceleration (24, 35) may be readily recorded. However, because these recorded metrics are stochastic, the greatest challenge for future research will be developing statistical analyses that can quantify climber exertion based on non-periodic metrics with a high degree of confidence. Embedded sensors will face this same challenge. But they face this challenge to a lesser degree. Force-derived climber metrics have been used extensively in understanding climber performance (36–44) (? ) in the sports science field. Metrics such as mean impulse force and the number of load changes are indicative of climber effort and skill. So, the challenges in using force-derived metrics from embedded sensors would be aligning their occurrence with movements, sequencing those movements, and selecting a suitable model for evaluating route difficulty. This is a multifaceted problem that will require future collaboration between researchers of different fields. The future success of the climber-centric approach will rely on the interdisciplinary work of sports scientists, engineers, computer scientists, and climbing gym owners. Quantifying human effort and skill performance is challenging in any athletic endeavor, and it is especially complicated for climbing. To generate and validate meaningful feature data that indicates climbing effort and skill, researchers in the fields of sports science, engineering, and computer science must work collaboratively to aid climbing gyms in producing accurately graded routes that satisfy their consumer base.

Determining the difficulty of a rock climbing route based on risk is relatively unexplored. the majority of work reported in this review aimed to determine route difficulty from technical systems, such as the V-Scale and Font Scale. Even those that did explore route difficulty determination with systems that incorporate risk, such as YDA and French Sport, the feature data for the models were only related to the technical grading of the route. There is an opportunity for future researchers to quantify risk as feature data in models. These feature data could be temperature, precipitation, humidity, number of protective gear, distance between protective gear, and height of the route. It is suspected that humidity would have a large impact on route difficulty because low humid conditions improve friction between the climbing and holds. Determining route difficulty with risk factors, and evaluating how the introduction of risk features affects model performance, would be an interesting development.

Selecting a machine and or deep learning model for future research in determining route difficulty is dependent on numerous factors. We recommend following these steps for selecting a suitable model:

1. Identify which rock climbing route grading system will be used.
2. Understand the factors affecting route grade determination within that system.
3. Formulate how to meaningfully quantify these factors with feature data. This is where collaboration with researchers in sports science and the rock climbing community is crucial.
4. After identifying these factors and how to quantify them, select whether your feature data falls within the Route-centric, Climber-centric, Path-finding and Path-generation approaches, or an entirely new approach.
5. Select a method (model) for your approach.

## 8 Conclusion

Climbers solve problems. These climbing route problems are defined by their difficulty grade, which is often times decided based on subjective, personal biases. In aims of quantifying climbing route difficulty with a more objective standard, researchers have implemented many machine and deep learning methods with varying success. This survey paper synthesized the state-of-the-art for determining rock climbing route difficulty with machine and deep learning; and, in doing so, has identified three main approaches: route-centric, climber-centric, and path finding and path generation. The most successful of these approaches used route-centric and path finding data with probabilistic and NLP-like methods. But this success was attained with standardized rock walls, which do not reflect the majority of walls in commercial climbing gyms. Future success in determining rock climbing difficulty in these chaotic environments likely rely on route-centric data extracted with computer vision and then fed through an NLP algorithm. Machine learning and deep learning methods keep evolving to solve route problems like climbers. With further evolution, these methods may solve the pervading Grading Bias Problem in determining rock climbing route difficulty.

## Footnotes

^1^Strength and technique are two different aspects of movement. For example, a climber with four points of contact (two hands and two feet) may shift most of his body weight to his left leg by bending the left knee and stretching the right leg straight. This would gauge technique. Now, when the climber seeks to move upward, the climber will unbend their left knee as the muscles of the left leg push upward. This would gauge the strength.

^2^MoonBoard 2016 is the only version that uses 142 holds. The 2017, 2019, and 2023 versions use all 198 holds.

^3^https://darth-grader.net.

^4^In the same work (23), it is observed that the perceived difficulty of a route has a positively accelerating, exponential relationship with route grade. It therefore becomes easier for climbers to *subjectively* distinguish between route difficulty at higher grades.
