# Corrigendum: Addressing grading bias in rock climbing: machine and deep learning approaches

**DOI:** 10.3389/fspor.2025.1570591

**Published:** 2025-02-20

**Authors:** B. O’Mara, M. S. Mahmud

**Affiliations:** Remote Sensing Laboratory, Department of Electrical and Computer Engineering, University of New Hampshire, Durham, NH, United States

**Keywords:** rock climbing, bouldering, route grade difficulty, deep learning, machine learning

A corrigendum on Addressing grading bias in rock climbing: machine and deep learning approaches O’Mara B, Mahmud MS. (2025). Addressing grading bias in rock climbing: machine and deep learning approaches. Front. Sports Act. Living 6:1512010. doi: 10.3389/fspor.2024.1512010

## Missing citation

In the published article. **[Dataset] Singh S. Rock. Climbing Gym Market Size, Share, Report, Forecast 2032 (2024)** was not cited in the article. The citation has now been inserted in **Introduction**, paragraph **2** and should read:

Climbing accessibility is highly dependent on route setters. Route setters produce climbing routes, the central service of a climbing gym. They are responsible for producing routes that are varied yet consistent in difficulty. Gyms vary their route difficulties to capture the largest audience possible (1), catering to a range of climber experience levels from novice to advanced. However, the grading scales used to rate climbing route difficulty are often subjective according to the region, the gym, and the setter of the route (2). General factors considered when determining route difficulty are rock hold types, the number of rock holds on a route, the distance between the rock holds, and the angle of ascent (3). Therefore, it seems that the positioning and sequencing of holds are critical to route difficulty. But holds may be positioned and sequenced in an almost infinite number of ways. Setting a route is like composing a song (4, 5); there are constraints that govern its composition, but the liberty to operate within those constraints is quite large. When operating within these constraints, a route can be developed in a multitude of ways. This wide variance of route generation is a challenge for generalizing route difficulty. without a large sample size, route setters introduce their own biases when determining route difficulty, which then inadvertently affects the climber (i.e., the customer).

The authors apologize for this error and state that this does not change the scientific conclusions of the article in any way. The original article has been updated.
